# Ruptured liver abscess presenting as pneumoperitoneum caused by *Klebsiella pneumoniae*: a case report

**DOI:** 10.1186/s12893-020-00858-w

**Published:** 2020-10-07

**Authors:** Thuong Pham Van, Son Vu Ngoc, Ngoc Anh Nguyen Hoang, Doan Hoang Huu, Tung Anh Dinh Duong

**Affiliations:** 1 Department of General Surgery, Haiphong University of Medicine and Pharmacy, Haiphong, Vietnam; 2 Pediatrics Department, Haiphong University of Medicine and Pharmacy, Haiphong, Vietnam

**Keywords:** Pneumoperitoneum, Ruptured liver abscess, *Klebsiella pneumoniae*, Case report

## Abstract

**Background:**

Spontaneous gas-forming pyogenic liver abscess (GFPLA) is a rare complication with a high fatality rate in spite of aggressive management. Clinical spectrum of GFPLA can mimic hollow viscus perforation as it usually accompanied by pneumoperitoneum and peritonitis. Up to now, GFPLA has not been well studied in Vietnam.

**Case presentation:**

We reported here a case with pneumoperitoneum caused by ruptured liver abscess in a 41-year-old man with a history of treated duodenal ulcer and uncontrolled type II diabetes mellitus. He had an epigastric pain associated with a high fever. Patient was diagnosed peritonitis and pneumoperitoneum presumed to be secondary to perforation of a hollow viscus and subjected to emergency laparotomy. We did not find any gastrointestinal perforation. Surprisingly, we detected a 4 cm × 4 cm pus-containing abscess in the left liver lobe of the liver. The abscess was ruptured. Pus was running into abdominal cavity through one hole. The abscess and abdominal cavities were cleaned up and abscess and abdominal drainages were performed. *K. pneumoniae* was isolated from culture of the abscess. The histopathological examination of the abscess did not yield any evidence of malignancy. Blood glucose levels were controlled. Antibiotic therapy was used according to antibiogram. A reassessment chest X-ray showed no air-fluid level or subdiaphragmatic air by the hospital day 14. Patient eventually made a full recovery and was discharged home 23 days after the operation.

**Conclusions:**

Ruptured GFPLA is a life-threatening complication. It is usually accompanied by peritonitis and pneumoperitoneum and can imitate hollow viscous perforation. In these cases, CT scan should be performed whenever it is possible to make a correct diagnosis. When the abscess has small size, partial hepatectomy might not be necessary and could be replaced by a careful cleaning and drainage of the abscess. Patient could show a good postoperative recovery following an appropriate antibiotic therapy.

## Background

Pyogenic liver abscess (PLA) is important cause of hospitalization and life threatening disease in low-middle income countries [6, 18]. Spontaneous gas-forming pyogenic liver abscess (GFPLA) is a rare complication with a high fatality rate in spite of aggressive management [3]. Clinical spectrum of GFPLA can mimic hollow viscus perforation as it usually accompanied by pneumoperitoneum and peritonitis [7]. Previous studies showed that *E. coli* and *K. pneumoniae* are the most common pathogens isolated in pyogenic liver abscess [10].

GFPLA is uncommon in western countries but often reported in Asian countries such as Taiwan and South Korea [5, 10]. Recently, ruptured liver abscess has received attention of clinicians and researchers in Vietnam [4, 17]. However, further studies are required in order to elucidate the general states and characteristics of GFPLA in this country where there is still a high percentage of low resource medical settings. Here, we reported a case of pneumoperitoneum due to ruptured pyogenic liver abscess caused by *Klebsiella pneumoniae*.

## Case presentation

We present a case of a 41-years-old man living in a rural area with a history of treated duodenal ulcer and uncontrolled type II diabetes mellitus. Five days before the hospital admission, this patient had an epigastric pain associated with a high fever (up to 39.5 °C). Four hours before the hospitalization, his epigastric pain was seriously and dramatically increased. A physical examination on admission to the emergency department revealed high temperature of 39 °C and generalized rigidity of the abdominal wall, suggesting a peritonitis. His heart rate was 109 beats/min and his blood pressure were 120/80 mmHg. There was not a clear clinical anemia or jaundice. His white blood cell count was 19.1 × 10^9^/L and the percentage of neutrophils was 85.3%. Other laboratory results were as follows: serum pro-calcitonin level, 17.73 ng/ml; serum glucose level, 14.7 mmol/L; serum bilirubin level, 37.6 mmol/L; serum aspartate aminotransferase level, 51 U/L and serum alanine aminotransferase level, 87 U/L.

**Fig. 1:**
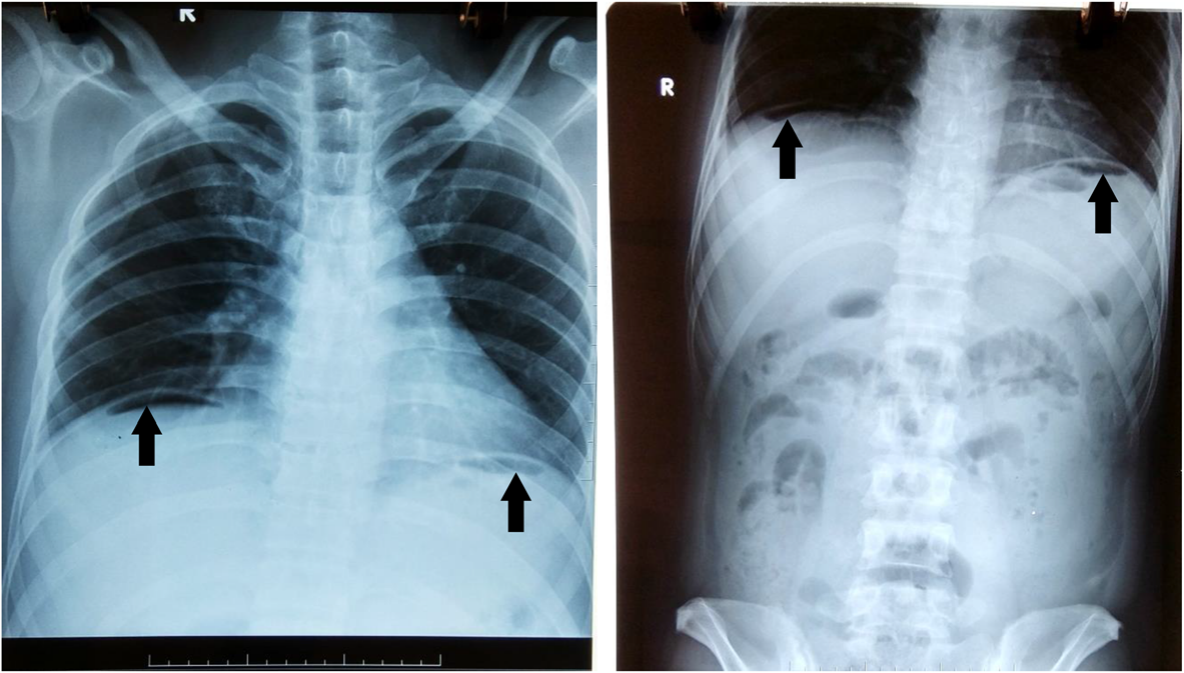
Gas under both domes of diaphragm on radiograph. Routine preoperative X-ray taken on admission showed free gas under both domes of diaphragm (arrows)

Both of the chest and abdominal X-ray showed bilateral subdiaphragmatic air, indicating pneumoperitoneum (Fig. 1). Consistently, ultrasound scan of the abdomen was conducted and detected free intraperitoneal air and fluid. It was hard to evaluate liver tissue due to the presence of intraperitoneal air and no hypoechoic foci was detected in the liver by this method. Unfortunately, computed tomography (CT) of the abdomen was not available by the time of the admission.

Patient was diagnosed peritonitis and pneumoperitoneum presumed to be secondary to perforation of a hollow viscus and treated for septic shock with intravenous fluid and broad-spectrum antibiotic. He was subjected to emergency laparotomy. Based on his previously reported duodenal ulcer history, we carefully checked and did not find any gastrointestinal perforation. Surprisingly, we detected a 4 cm × 4 cm pus-containing abscess in the left liver lobe of the liver. The abscess was ruptured. Pus was running into abdominal cavity through one hole. At the end of the operation, 80 ml of turbid pus was drained from the subphrenic and subhepatic spaces. We carefully checked and did not find any underlying disease of the biliary tract. We concluded that the pneumoperitoneum resulted from spontaneous rupture of the hepatic abscess in the left liver. We placed abdominal drains in the abscess cavity and the subhepatic area. *K. pneumoniae* was isolated from culture of the abscess. The histopathological examination of the abscess did not yield any evidence of malignancy. Blood glucose levels were controlled. Antibiotic therapy was used according to antibiogram (piperacilin and tazobactam). A reassessment chest X-ray showed no air-fluid level or subdiaphragmatic air by the hospital day 14. The patient eventually made a full recovery without any complication and was discharged home 23 days after the operation.

## Discussion and conclusions

Pyogenic liver abscess (PLA) is a common infectious disease worldwide relating to a mortality rate ranging between 15 and 19% [15, 18]. Gas-forming pyogenic liver abscess (GFPLA) remains one of the most dangerous complication with a high fatality rate in spite of aggressive management [10]. *Klebsiella pneumoniae* is considered to surpass *Escherichia coli (E. coli)* to become the major pathogen of pyogenic liver abscesses, especially in GFPLA and in patients with diabetic mellitus (DM) [9, 10]. Here, we reported a case of *Klebsiella pneumoniae-*induced gas-forming pyogenic liver abscess with uncontrolled DM history.

CT-Scan plays a key role in the diagnosis of gas-forming pyogenic liver abscess [4, 12]. Unfortunately, there is a fact that access to advanced imaging systems like CT is still limited in developing countries. In our case, CT was not available by the time of hospital admission of the patient. But this was not the unique reason for the inaccurate preoperative diagnosis as pneumoperitoneum presumed to be secondary to perforation of a hollow viscus. Indeed, there are several other causes such as: history of duodenal ulcer, the relatively small size of this liver abscess, and the deficiency of ultrasound scan in case of ruptured liver abscess. By presenting this case of GFPLA, we aimed to highlight the importance of careful examination by the surgeons when abdominal peritonitis and pneumoperitoneum were present without any perforation of a hollow viscus. This case also implied that CT scan should be performed wherever it is possible to make an early and correct diagnosis of GFPLA which in turn, help to decrease the operative time and improve patient outcomes.

Several studies have reported the cases of successful non-surgical treatment of ruptured pyogenic liver abscess [11, 13]. In these cases, patients with ruptured liver abscess associated with septic shock and high glycemic level can be successfully treated with percutaneous ultrasound-guided drainage associated with IV broad spectrum antibiotics without surgical intervention. However, these cases had prolonged hospital stay (40–52 days). According to a study of Shiba H., in case of ruptured liver abscess associated with peritonitis and sepsis, drain abscess should not be performed. Instead of that, they cut the part of liver which contained abscess and cleaned the abdominal cavity. Their patient recovered well after surgery without any complication and discharged at the postoperative day 30 [16]. In our case, partial hepatectomy was not conducted to remove the abscess. Alternatively, we firstly performed cleaning of the abscess cavity and removed all the pus inside, in parallel with cleaning of the abdominal cavity. After that, one of the abdominal drainages was directly put inside the abscess. Our patient presented a good recovery without any complication and he was discharged at the 23rd postoperative day. We suggest that this method might be useful for treating ruptured PLA with a single and relatively small size abscess.

In our case, emergency laparotomy was chosen as the first choice following the diagnosis of a secondary peritonitis in order to carry out a careful abdominal lavage. This method also allowed the surgeons to perform cleaning of the abscess cavity and contributed to the successful treatment without partial hepatectomy. Laparoscopy lavage is an alternative option in the treatment of purulent peritonitis but it is associated with a significantly higher rate of reoperations and a higher rate of intra-abdominal abscess [1]. Furthermore, laparoscopic surgery and training is not widely available in the developing countries. Therefore, we suggest that laparotomy might be suitable to combine with the treatment of rupture liver abscess using abdominal drainages.

Finally, *K. pneumoniae* PLA is predominantly seen in Southeast Asia but only few cases have been reported in Vietnam [2, 4]. Strikingly, current researches showed that *K. pneumoniae* infection is becoming an emerging public health problem in Vietnam due to its carbapenem-resistance [8, 14]. Therefore, further investigations are required to clarify the clinical and paraclinical characteristics of *K. pneumoniae* PLA in Vietnam.

In conclusion, ruptured gas-containing PLA is a life-threatening complication. It is usually accompanied by peritonitis and pneumoperitoneum and can imitate hollow viscous perforation. In these cases, CT scan should be performed whenever it is possible to make a correct diagnosis. When the abscess has small size, partial hepatectomy might not be necessary and could be replaced by a careful cleaning and drainage of the abscess in association with an appropriate antibiotic therapy.

## Data Availability

All data generated or analyzed during this study are included in this published article.

## Abbreviations

CT: Computed tomography
GFPLA: Gas-forming pyogenic liver abscess
IV: Intravenous
PLA: Pyogenic liver abscess
